# Supplementary material for: Voxel-Wise Comparison of Co-Registered Quantitative CT and Hyperpolarised Gas Diffusion-Weighted MRI Measurements in IPF
Source: Diagnostics (Basel). 2023 Nov 21;13(23):3497. doi: 10.3390/diagnostics13233497 (PMC10706152; doi:10.3390/diagnostics13233497)
Supplement: Supplementary file 1 [file diagnostics-13-03497-s001.zip › Table S1.pdf]

Supplementary Table S1: Summary of imaging metrics for each individual IPF patient at baseline. Metrics include: usual interstitial pneumonia (UIP) pattern on volumetric CT as visually assessed by thoracic radiologists during IPF diagnosis, Dice overlap coefficients, CALIPER pattern percentages, and ^{3}He diffusion-weighted MRI metrics. Non-involved = normal physiological lung; hyperlucent = emphysematous lung.

	Visual CT UIP pattern	Dice Coefficients			CALIPER patterns %					^{3}He diffusion-weighted MRI		
		CT vs ^{1}H	^{3}He ventilation vs diffusion	^{3}He diffusion vs CT	Non-involved	Ground Glass	Reticular	Honeycomb	Hyperlucent	^{3}He ADC (cm^2/s)	^{3}He Lm_D (μm)	Abnormal ^{3}He Lm_D % (>406 μm)
IPF01	Definite	0.956	0.910	0.906	51.2	37.6	11	0.1	0	0.358	396	41.7
IPF02	Probable	0.952	0.913	0.918	95.2	0.6	0.5	0.04	3.7	0.261	342	16.1
IPF03	Probable	0.955	0.934	0.930	83	12.4	4.5	0.01	0	0.307	370	30.3
IPF04	Definite	0.961	0.933	0.937	68.2	13.7	18	0.1	0	0.326	388	38.1
IPF05	Probable	0.949	0.917	0.906	91.8	1.1	0.3	0.03	6.7	0.335	391	42.1
IPF06	Probable	0.956	0.937	0.927	58.9	34.4	6.6	0.01	0	0.292	358	19.3
IPF07	Definite	0.939	0.911	0.906	81.7	14.5	3.6	0.2	0	0.255	333	13.4
IPF08	Definite	0.968	0.927	0.930	67.8	21.4	3.6	3.3	4	0.481	450	69.6
IPF09	Probable	0.957	0.926	0.924	94.1	1.2	0.6	0	4.2	0.247	332	8.9
IPF10	Definite	0.954	0.919	0.922	79.7	15.4	4.7	0.2	0.1	0.363	399	42.1
IPF11	Probable	0.956	0.922	0.919	82.9	8.4	2	1.3	5.3	0.445	443	67.3
IPF12	Definite	0.964	0.936	0.935	76.2	19.3	4.1	0.3	0.1	0.403	428	59.7
IPF13	Definite	0.940	0.910	0.890	54.3	18.5	24.7	2.4	0.1	0.425	422	55.1
IPF14	Probable	0.948	0.918	0.912	91.3	5.4	1.8	0.01	1.6	0.294	368	24.6
IPF15	Probable	0.959	0.925	0.930	89.5	4.9	2.3	0.05	3.3	0.350	405	48.8
IPF16	Probable	0.954	0.920	0.925	87.1	0	1.5	0.03	11.4	0.222	312	4.9
All		0.954	0.922	0.920	78.9	12.4	5.5	0.5	2.7	0.335	384	36.8
