# Supplementary figures and images for: Voxel-Wise Comparison of Co-Registered Quantitative CT and Hyperpolarised Gas Diffusion-Weighted MRI Measurements in IPF
Source: Diagnostics (Basel). 2023 Nov 21;13(23):3497. doi: 10.3390/diagnostics13233497 (PMC10706152; doi:10.3390/diagnostics13233497)

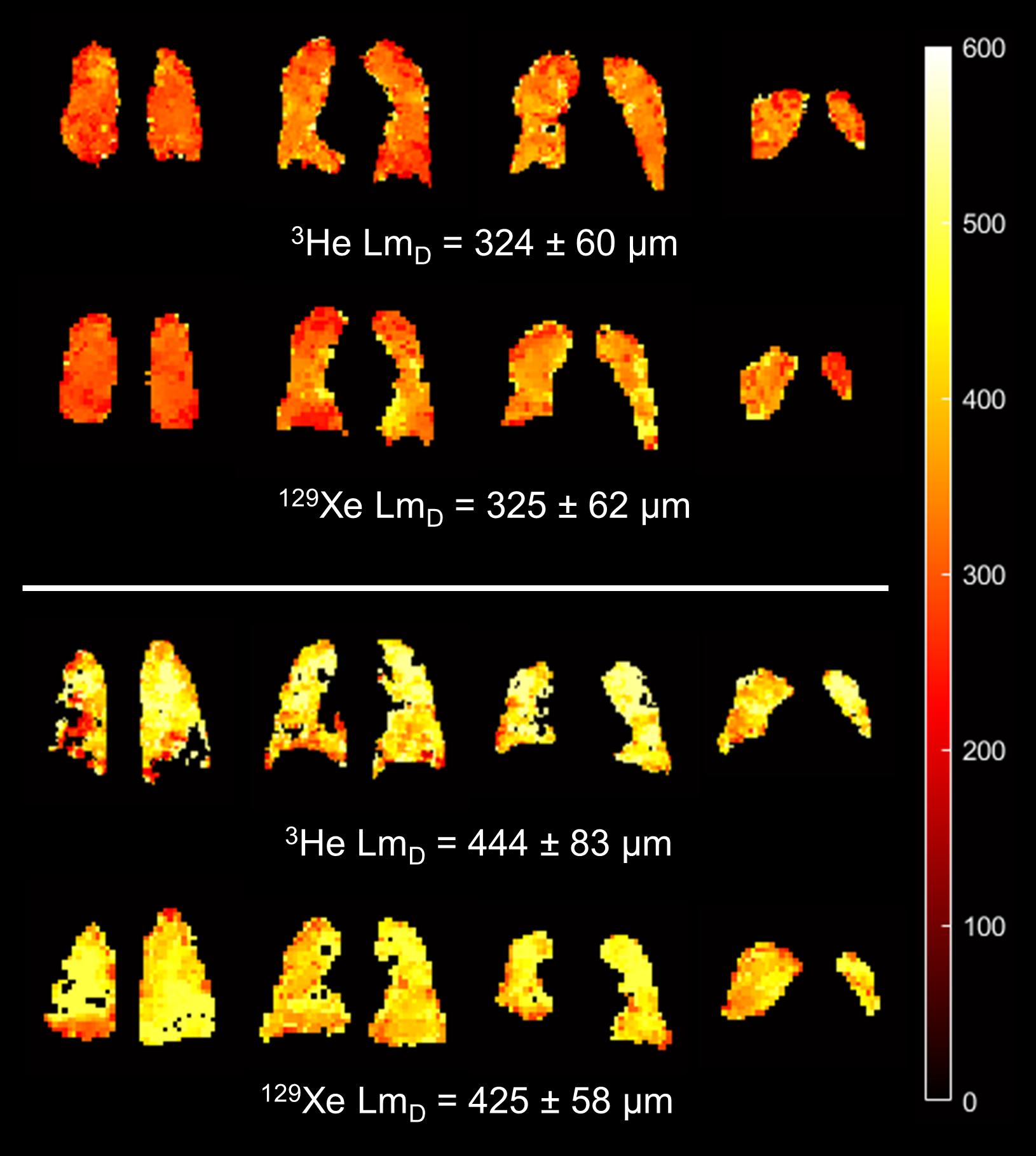

Supplement: Supplementary file 1 [file diagnostics-13-03497-s001.zip › Figure S1.tif]
